# *cis*-Aqua­bromidobis(di-2-pyridyl­amine-κ^2^ *N*,*N*′)manganese(II) bromide

**DOI:** 10.1107/S1600536811048100

**Published:** 2011-11-19

**Authors:** Kwang Ha

**Affiliations:** a School of Applied Chemical Engineering, The Research Institute of Catalysis, Chonnam National University, Gwangju 500-757, Republic of Korea

## Abstract

In the title compound, [MnBr(C_10_H_9_N_3_)_2_(H_2_O)]Br, the Mn^II^ ion is six-coordinated in a considerably distorted *cis*-N_4_BrO octa­hedral environment defined by four N atoms of two chelating di-2-pyridyl­amine (dpa) ligands, one Br^−^ anion and one O atom of a water ligand. As a result of the different *trans* effects of Br, N and O atoms, the Mn—N bond *trans* to the Br atom is slightly longer than the Mn—N bond *trans* to the N or O atoms. In the crystal, the dpa ligands are not planar, the dihedral angles between the two pyridine rings being 29.2 (4) and 28.2 (3)°. The complex cations and the Br^−^ anions are linked by inter­molecular O—H⋯Br and N—H⋯Br hydrogen bonds. Inter­molecular π–π inter­actions are present between the pyridine rings, with a centroid–centroid distance of 3.793 (4) Å.

## Related literature

For the structures of related Mn^II^ complexes with a di-2-pyridyl­amine ligand, see: Bose *et al.* (2005 ▶).
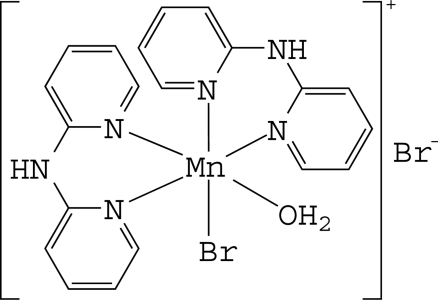


## Experimental

### 

#### Crystal data

[MnBr(C_10_H_9_N_3_)_2_(H_2_O)]Br
*M* *_r_* = 575.18
Triclinic, 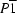

*a* = 8.3990 (15) Å
*b* = 10.0022 (18) Å
*c* = 13.613 (2) Å
α = 90.692 (4)°
β = 103.619 (4)°
γ = 98.556 (4)°
*V* = 1097.8 (3) Å^3^
*Z* = 2
Mo *K*α radiation
μ = 4.27 mm^−1^
*T* = 200 K
0.22 × 0.21 × 0.19 mm

#### Data collection

Bruker SMART 1000 CCD diffractometer
Absorption correction: multi-scan (*SADABS*; Bruker, 2001 ▶) *T*_min_ = 0.708, *T*_max_ = 1.000
6807 measured reflections
4215 independent reflections
2569 reflections with *I* > 2σ(*I*)
*R*_int_ = 0.049

#### Refinement

*R*[*F* ^2^ > 2σ(*F* ^2^)] = 0.057
*wR*(*F* ^2^) = 0.174
*S* = 0.96
4215 reflections
271 parameters
H-atom parameters constrained
Δρ_max_ = 1.02 e Å^−3^
Δρ_min_ = −1.02 e Å^−3^

### 

Data collection: *SMART* (Bruker, 2007 ▶); cell refinement: *SAINT* (Bruker, 2007 ▶); data reduction: *SAINT*; program(s) used to solve structure: *SHELXS97* (Sheldrick, 2008 ▶); program(s) used to refine structure: *SHELXL97* (Sheldrick, 2008 ▶); molecular graphics: *ORTEP-3* (Farrugia, 1997 ▶) and *PLATON* (Spek, 2009 ▶); software used to prepare material for publication: *SHELXL97*.

## Supplementary Material

Crystal structure: contains datablock(s) global, I. DOI: 10.1107/S1600536811048100/hy2487sup1.cif

Structure factors: contains datablock(s) I. DOI: 10.1107/S1600536811048100/hy2487Isup2.hkl

Additional supplementary materials:  crystallographic information; 3D view; checkCIF report

## Figures and Tables

**Table 1:** Selected bond lengths (Å)

Mn1—O1	2.154 (6)
Mn1—N1	2.318 (6)
Mn1—N3	2.256 (5)
Mn1—N4	2.246 (6)
Mn1—N6	2.266 (6)
Mn1—Br1	2.6395 (13)

**Table 2:** Hydrogen-bond geometry (Å, °)

*D*—H⋯*A*	*D*—H	H⋯*A*	*D*⋯*A*	*D*—H⋯*A*
O1—H1*A*⋯Br2^i^	0.84	2.50	3.304 (5)	160
O1—H1*B*⋯Br1^ii^	0.84	2.44	3.272 (5)	171
N2—H2*N*⋯Br2^iii^	0.92	2.62	3.472 (6)	154
N5—H5*N*⋯Br2^iv^	0.92	2.63	3.503 (6)	159

Symmetry codes: (i) 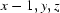; (ii) 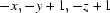; (iii) 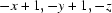; (iv) 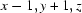.

**Fig. 1.**
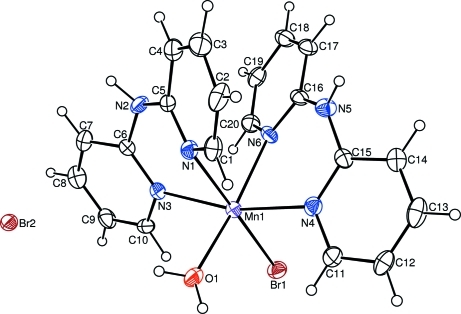
The molecular structure of the title compound, with displacement ellipsoids drawn at the 30% probability level.

**Fig. 2.**
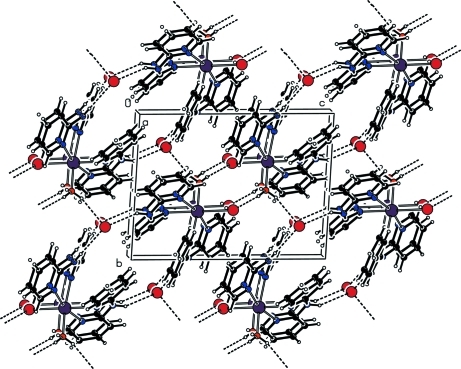
View of the crystal packing of the title compound. Hydrogen bonds are drawn with dashed lines.

## supplementary crystallographic information

### Comment

Cationic Mn^II^ complexes of di-2-pyridylamine (dpa; C_10_H_9_N_3_) ligand, such as [Mn*X*(dpa)_2_(H_2_O)]ClO_4_ (*X* = N_3_^-^, NCO^-^), have been investigated previously (Bose *et al.*, 2005).

The asymmetric unit of the title compound, [MnBr(dpa)_2_(H_2_O)]Br, consists of a cationic Mn^II^ complex and a Br^-^ anion (Fig. 1). In the complex, the Mn^II^ ion is six-coordinated in a considerably distorted *cis*-N_4_BrO octahedral environment defined by four N atoms of two chelating dpa ligands, one Br^-^ anion and one O atom of a water ligand. The main contribution to the distortion is the tight N—Mn—N chelating angles, which results in non-linear *trans* axes [N3—Mn1—N4 = 165.8 (2) and O1—Mn1—N6 = 171.6 (2)°]. But, the apical Br1—Mn1—N1 bond is almost linear with a bond angle of 177.25 (15)°. The Mn—N(dpa) bond lengths are slightly different and longer than the Mn—O(H_2_O) bond (Table 1). As a result of the different *trans* effects of Br, N and O atoms, the Mn—N bond *trans* to the Br atom is somewhat longer than the Mn—N bond *trans* to the N or O atom. In the crystal structure, the dpa ligands are not planar. The dihedral angles between the two pyridine rings of dpa are 29.2 (4) and 28.2 (3)°. The complexes are stacked in columns along the *a* axis, and the components are linked by intermolecular O—H···Br and N—H···Br hydrogen bonds (Fig. 2, Table 2). Intermolecular π–π interactions between the pyridine rings are present, with a centroid–centroid distance of 3.793 (4) Å.

### Experimental

To a solution of MnBr_2_.4H_2_O (0.2882 g, 1.005 mmol) in EtOH (30 ml) was added di-2-pyridylamine (0.3465 g, 2.024 mmol) and stirred for 3 h at room temperature. The formed precipitate was separated by filtration and washed with EtOH and acetone and dried at 50°C to give a white powder (0.4092 g). Crystals suitable for X-ray analysis were obtained by slow evaporation from a CH_3_NO_2_/MeOH solution.

### Refinement

C-bound H atoms were positioned geometrically and allowed to ride on their respective parent atoms [C—H = 0.95 Å and *U*_iso_(H) = 1.2*U*_eq_(C)]. N- and O-bound H atoms were located from difference Fourier maps and allowed to ride on their parent atoms in the final cycles of refinement, with N—H = 0.92, O—H = 0.84 Å and *U*_iso_(H) = 1.5*U*_eq_(N, O). The highest peak (1.02 e Å^-3^) and the deepest hole (-1.02 e Å^-3^) in the difference Fourier map are located 1.19 and 0.94 Å from atoms Br2 and Br1, respectively.

### Crystal data

**Table:**

[MnBr(C_10_H_9_N_3_)_2_(H_2_O)]Br	*Z* = 2
*M**_r_* = 575.18	*F*(000) = 570
Triclinic, *P*1	*D*_x_ = 1.740 Mg m^−^^3^
Hall symbol: -P 1	Mo *K*α radiation, λ = 0.71073 Å
*a* = 8.3990 (15) Å	Cell parameters from 2405 reflections
*b* = 10.0022 (18) Å	θ = 2.5–25.9°
*c* = 13.613 (2) Å	µ = 4.27 mm^−^^1^
α = 90.692 (4)°	*T* = 200 K
β = 103.619 (4)°	Block, colorless
γ = 98.556 (4)°	0.22 × 0.21 × 0.19 mm
*V* = 1097.8 (3) Å^3^	

### Data collection

**Table:**

Bruker SMART 1000 CCD diffractometer	4215 independent reflections
Radiation source: fine-focus sealed tube	2569 reflections with *I* > 2σ(*I*)
graphite	*R*_int_ = 0.049
φ and ω scans	θ_max_ = 26.0°, θ_min_ = 2.1°
Absorption correction: multi-scan (*SADABS*; Bruker, 2001)	*h* = −10→9
*T*_min_ = 0.708, *T*_max_ = 1.000	*k* = −12→10
6807 measured reflections	*l* = −13→16

### Refinement

**Table:**

Refinement on *F*^2^	Primary atom site location: structure-invariant direct methods
Least-squares matrix: full	Secondary atom site location: difference Fourier map
*R*[*F*^2^ > 2σ(*F*^2^)] = 0.057	Hydrogen site location: inferred from neighbouring sites
*wR*(*F*^2^) = 0.174	H-atom parameters constrained
*S* = 0.96	*w* = 1/[σ^2^(*F*_o_^2^) + (0.0886*P*)^2^] where *P* = (*F*_o_^2^ + 2*F*_c_^2^)/3
4215 reflections	(Δ/σ)_max_ < 0.001
271 parameters	Δρ_max_ = 1.02 e Å^−^^3^
0 restraints	Δρ_min_ = −1.02 e Å^−^^3^

### Fractional atomic coordinates and isotropic or equivalent isotropic displacement parameters (Å^2^)

**Table:**

	*x*	*y*	*z*	*U*_iso_*/*U*_eq_	
Mn1	0.07207 (13)	0.66748 (11)	0.32514 (7)	0.0313 (3)	
Br1	0.27645 (10)	0.65301 (8)	0.50321 (5)	0.0403 (3)	
O1	−0.0809 (7)	0.4759 (5)	0.3301 (4)	0.0525 (15)	
H1A	−0.0981	0.4271	0.2769	0.079*	
H1B	−0.1391	0.4383	0.3674	0.079*	
N1	−0.0969 (7)	0.6794 (6)	0.1652 (4)	0.0330 (14)	
N2	0.1133 (7)	0.6848 (6)	0.0779 (4)	0.0372 (15)	
H2N	0.1256	0.6768	0.0128	0.056*	
N3	0.2136 (7)	0.5727 (6)	0.2284 (4)	0.0329 (14)	
N4	−0.0788 (7)	0.7969 (6)	0.3899 (4)	0.0339 (14)	
N5	−0.0214 (7)	0.9888 (6)	0.2943 (4)	0.0366 (15)	
H5N	−0.0951	1.0440	0.2639	0.055*	
N6	0.2054 (7)	0.8730 (5)	0.2987 (4)	0.0327 (14)	
C1	−0.2490 (9)	0.7091 (8)	0.1666 (5)	0.0403 (19)	
H1	−0.3009	0.6705	0.2168	0.048*	
C2	−0.3311 (11)	0.7904 (9)	0.1005 (6)	0.051 (2)	
H2	−0.4397	0.8052	0.1018	0.062*	
C3	−0.2485 (11)	0.8519 (9)	0.0298 (6)	0.053 (2)	
H3	−0.2951	0.9178	−0.0128	0.064*	
C4	−0.1021 (11)	0.8160 (8)	0.0235 (6)	0.047 (2)	
H4	−0.0490	0.8519	−0.0270	0.057*	
C5	−0.0286 (9)	0.7255 (7)	0.0914 (5)	0.0341 (17)	
C6	0.2109 (9)	0.5943 (7)	0.1305 (5)	0.0319 (16)	
C7	0.3075 (9)	0.5330 (8)	0.0785 (6)	0.0402 (19)	
H7	0.2981	0.5444	0.0083	0.048*	
C8	0.4167 (10)	0.4554 (8)	0.1323 (6)	0.050 (2)	
H8	0.4865	0.4150	0.0992	0.060*	
C9	0.4269 (10)	0.4354 (8)	0.2317 (6)	0.044 (2)	
H9	0.5049	0.3840	0.2690	0.053*	
C10	0.3202 (9)	0.4921 (7)	0.2772 (5)	0.0377 (18)	
H10	0.3216	0.4738	0.3456	0.045*	
C11	−0.1581 (10)	0.7396 (8)	0.4578 (5)	0.0417 (19)	
H11	−0.1418	0.6507	0.4770	0.050*	
C12	−0.2624 (9)	0.8039 (9)	0.5012 (6)	0.043 (2)	
H12	−0.3206	0.7591	0.5464	0.052*	
C13	−0.2780 (10)	0.9345 (10)	0.4761 (6)	0.050 (2)	
H13	−0.3464	0.9823	0.5054	0.060*	
C14	−0.1948 (8)	0.9969 (8)	0.4084 (5)	0.0361 (18)	
H14	−0.2041	1.0880	0.3922	0.043*	
C15	−0.0972 (8)	0.9252 (7)	0.3641 (5)	0.0322 (17)	
C16	0.1330 (9)	0.9824 (7)	0.2764 (5)	0.0346 (17)	
C17	0.2081 (10)	1.0955 (8)	0.2350 (5)	0.0398 (19)	
H17	0.1530	1.1715	0.2185	0.048*	
C18	0.3648 (10)	1.0939 (8)	0.2186 (5)	0.0429 (19)	
H18	0.4179	1.1682	0.1893	0.051*	
C19	0.4427 (9)	0.9823 (8)	0.2456 (5)	0.0402 (19)	
H19	0.5505	0.9792	0.2360	0.048*	
C20	0.3607 (9)	0.8760 (8)	0.2867 (5)	0.0376 (18)	
H20	0.4161	0.8013	0.3076	0.045*	
Br2	0.78643 (10)	0.23349 (8)	0.14966 (5)	0.0421 (3)	

### Atomic displacement parameters (Å^2^)

**Table:**

	*U*^11^	*U*^22^	*U*^33^	*U*^12^	*U*^13^	*U*^23^
Mn1	0.0396 (7)	0.0264 (6)	0.0306 (6)	0.0082 (5)	0.0117 (5)	0.0025 (4)
Br1	0.0467 (5)	0.0432 (5)	0.0329 (4)	0.0121 (4)	0.0100 (3)	0.0078 (3)
O1	0.081 (4)	0.044 (3)	0.036 (3)	−0.009 (3)	0.031 (3)	−0.003 (2)
N1	0.036 (3)	0.031 (3)	0.031 (3)	0.001 (3)	0.008 (3)	−0.003 (3)
N2	0.049 (4)	0.045 (4)	0.023 (3)	0.018 (3)	0.014 (3)	0.005 (3)
N3	0.041 (4)	0.027 (3)	0.033 (3)	0.010 (3)	0.010 (3)	0.001 (2)
N4	0.036 (3)	0.035 (4)	0.030 (3)	0.006 (3)	0.005 (3)	−0.001 (3)
N5	0.032 (3)	0.040 (4)	0.039 (3)	0.014 (3)	0.007 (3)	0.010 (3)
N6	0.037 (4)	0.022 (3)	0.037 (3)	0.008 (3)	0.004 (3)	0.002 (2)
C1	0.034 (4)	0.046 (5)	0.038 (4)	0.003 (4)	0.005 (3)	−0.013 (4)
C2	0.048 (5)	0.073 (7)	0.034 (4)	0.021 (5)	0.004 (4)	−0.011 (4)
C3	0.063 (6)	0.055 (6)	0.043 (5)	0.034 (5)	0.001 (4)	0.004 (4)
C4	0.064 (6)	0.043 (5)	0.038 (4)	0.019 (4)	0.011 (4)	−0.002 (4)
C5	0.051 (5)	0.028 (4)	0.025 (3)	0.013 (3)	0.008 (3)	0.000 (3)
C6	0.040 (4)	0.024 (4)	0.032 (4)	0.005 (3)	0.009 (3)	0.001 (3)
C7	0.041 (5)	0.045 (5)	0.036 (4)	0.006 (4)	0.012 (3)	−0.008 (3)
C8	0.048 (5)	0.045 (5)	0.056 (5)	0.010 (4)	0.009 (4)	−0.007 (4)
C9	0.049 (5)	0.033 (5)	0.052 (5)	0.018 (4)	0.009 (4)	0.001 (4)
C10	0.054 (5)	0.031 (4)	0.035 (4)	0.012 (4)	0.020 (4)	0.009 (3)
C11	0.053 (5)	0.041 (5)	0.031 (4)	0.010 (4)	0.008 (4)	0.001 (3)
C12	0.035 (4)	0.063 (6)	0.037 (4)	0.010 (4)	0.017 (3)	−0.004 (4)
C13	0.034 (5)	0.075 (7)	0.042 (5)	0.023 (4)	0.004 (4)	−0.014 (4)
C14	0.025 (4)	0.042 (5)	0.038 (4)	0.007 (3)	0.001 (3)	−0.006 (3)
C15	0.030 (4)	0.030 (4)	0.033 (4)	0.009 (3)	−0.001 (3)	0.000 (3)
C16	0.045 (5)	0.026 (4)	0.027 (4)	0.008 (3)	−0.007 (3)	0.004 (3)
C17	0.051 (5)	0.031 (4)	0.034 (4)	0.013 (4)	0.001 (4)	0.008 (3)
C18	0.051 (5)	0.029 (4)	0.041 (4)	0.002 (4)	0.000 (4)	0.004 (3)
C19	0.031 (4)	0.056 (5)	0.032 (4)	0.002 (4)	0.007 (3)	−0.004 (4)
C20	0.036 (4)	0.038 (5)	0.038 (4)	0.011 (4)	0.006 (3)	0.003 (3)
Br2	0.0507 (5)	0.0409 (5)	0.0388 (4)	0.0156 (4)	0.0133 (4)	0.0053 (3)

### Geometric parameters (Å, °)

**Table:**

Mn1—O1	2.154 (6)	C3—H3	0.9500
Mn1—N1	2.318 (6)	C4—C5	1.405 (10)
Mn1—N3	2.256 (5)	C4—H4	0.9500
Mn1—N4	2.246 (6)	C6—C7	1.394 (10)
Mn1—N6	2.266 (6)	C7—C8	1.375 (10)
Mn1—Br1	2.6395 (13)	C7—H7	0.9500
O1—H1A	0.8400	C8—C9	1.354 (11)
O1—H1B	0.8400	C8—H8	0.9500
N1—C5	1.324 (9)	C9—C10	1.383 (10)
N1—C1	1.358 (8)	C9—H9	0.9500
N2—C5	1.366 (9)	C10—H10	0.9500
N2—C6	1.403 (8)	C11—C12	1.390 (10)
N2—H2N	0.9200	C11—H11	0.9500
N3—C6	1.347 (8)	C12—C13	1.372 (12)
N3—C10	1.356 (9)	C12—H12	0.9500
N4—C11	1.347 (10)	C13—C14	1.384 (12)
N4—C15	1.356 (9)	C13—H13	0.9500
N5—C15	1.375 (9)	C14—C15	1.396 (10)
N5—C16	1.385 (9)	C14—H14	0.9500
N5—H5N	0.9200	C16—C17	1.402 (11)
N6—C16	1.334 (8)	C17—C18	1.388 (11)
N6—C20	1.348 (9)	C17—H17	0.9500
C1—C2	1.356 (10)	C18—C19	1.387 (10)
C1—H1	0.9500	C18—H18	0.9500
C2—C3	1.410 (12)	C19—C20	1.376 (11)
C2—H2	0.9500	C19—H19	0.9500
C3—C4	1.353 (11)	C20—H20	0.9500
			
O1—Mn1—N4	97.1 (2)	N1—C5—N2	121.2 (6)
O1—Mn1—N3	91.0 (2)	N1—C5—C4	120.8 (7)
N4—Mn1—N3	165.8 (2)	N2—C5—C4	118.0 (7)
O1—Mn1—N6	171.6 (2)	N3—C6—C7	122.5 (6)
N4—Mn1—N6	81.6 (2)	N3—C6—N2	120.3 (6)
N3—Mn1—N6	88.6 (2)	C7—C6—N2	117.2 (6)
O1—Mn1—N1	85.7 (2)	C8—C7—C6	117.6 (7)
N4—Mn1—N1	89.9 (2)	C8—C7—H7	121.2
N3—Mn1—N1	79.1 (2)	C6—C7—H7	121.2
N6—Mn1—N1	86.0 (2)	C9—C8—C7	121.4 (7)
O1—Mn1—Br1	95.45 (15)	C9—C8—H8	119.3
N4—Mn1—Br1	92.44 (14)	C7—C8—H8	119.3
N3—Mn1—Br1	98.39 (14)	C8—C9—C10	118.0 (7)
N6—Mn1—Br1	92.92 (14)	C8—C9—H9	121.0
N1—Mn1—Br1	177.25 (15)	C10—C9—H9	121.0
Mn1—O1—H1A	112.9	N3—C10—C9	123.0 (7)
Mn1—O1—H1B	138.1	N3—C10—H10	118.5
H1A—O1—H1B	108.5	C9—C10—H10	118.5
C5—N1—C1	118.4 (6)	N4—C11—C12	123.4 (8)
C5—N1—Mn1	119.0 (5)	N4—C11—H11	118.3
C1—N1—Mn1	113.4 (4)	C12—C11—H11	118.3
C5—N2—C6	131.1 (6)	C13—C12—C11	117.4 (8)
C5—N2—H2N	117.9	C13—C12—H12	121.3
C6—N2—H2N	103.9	C11—C12—H12	121.3
C6—N3—C10	117.4 (6)	C12—C13—C14	120.4 (7)
C6—N3—Mn1	127.2 (4)	C12—C13—H13	119.8
C10—N3—Mn1	115.3 (4)	C14—C13—H13	119.8
C11—N4—C15	118.9 (6)	C13—C14—C15	119.6 (7)
C11—N4—Mn1	116.3 (5)	C13—C14—H14	120.2
C15—N4—Mn1	124.8 (5)	C15—C14—H14	120.2
C15—N5—C16	130.2 (6)	N4—C15—N5	121.7 (6)
C15—N5—H5N	103.2	N4—C15—C14	120.4 (7)
C16—N5—H5N	126.5	N5—C15—C14	117.9 (7)
C16—N6—C20	118.2 (7)	N6—C16—N5	120.6 (7)
C16—N6—Mn1	124.7 (5)	N6—C16—C17	122.3 (7)
C20—N6—Mn1	115.9 (4)	N5—C16—C17	117.1 (6)
C2—C1—N1	123.8 (8)	C18—C17—C16	118.5 (7)
C2—C1—H1	118.1	C18—C17—H17	120.7
N1—C1—H1	118.1	C16—C17—H17	120.7
C1—C2—C3	117.2 (8)	C19—C18—C17	119.0 (7)
C1—C2—H2	121.4	C19—C18—H18	120.5
C3—C2—H2	121.4	C17—C18—H18	120.5
C4—C3—C2	119.1 (8)	C20—C19—C18	118.7 (7)
C4—C3—H3	120.4	C20—C19—H19	120.6
C2—C3—H3	120.4	C18—C19—H19	120.6
C3—C4—C5	120.0 (8)	N6—C20—C19	123.0 (7)
C3—C4—H4	120.0	N6—C20—H20	118.5
C5—C4—H4	120.0	C19—C20—H20	118.5
			
O1—Mn1—N1—C5	−139.0 (5)	Mn1—N1—C5—C4	−137.2 (6)
N4—Mn1—N1—C5	123.8 (5)	C6—N2—C5—N1	0.7 (11)
N3—Mn1—N1—C5	−47.1 (5)	C6—N2—C5—C4	−178.1 (7)
N6—Mn1—N1—C5	42.2 (5)	C3—C4—C5—N1	−3.2 (11)
O1—Mn1—N1—C1	74.7 (5)	C3—C4—C5—N2	175.6 (7)
N4—Mn1—N1—C1	−22.4 (5)	C10—N3—C6—C7	2.7 (10)
N3—Mn1—N1—C1	166.6 (5)	Mn1—N3—C6—C7	178.1 (6)
N6—Mn1—N1—C1	−104.1 (5)	C10—N3—C6—N2	−175.0 (7)
O1—Mn1—N3—C6	111.4 (6)	Mn1—N3—C6—N2	0.4 (10)
N4—Mn1—N3—C6	−13.8 (13)	C5—N2—C6—N3	−26.7 (11)
N6—Mn1—N3—C6	−60.2 (6)	C5—N2—C6—C7	155.5 (7)
N1—Mn1—N3—C6	26.0 (6)	N3—C6—C7—C8	−4.5 (12)
Br1—Mn1—N3—C6	−153.0 (6)	N2—C6—C7—C8	173.2 (7)
O1—Mn1—N3—C10	−73.1 (5)	C6—C7—C8—C9	2.1 (13)
N4—Mn1—N3—C10	161.6 (8)	C7—C8—C9—C10	1.9 (13)
N6—Mn1—N3—C10	115.3 (5)	C6—N3—C10—C9	1.6 (11)
N1—Mn1—N3—C10	−158.5 (6)	Mn1—N3—C10—C9	−174.3 (6)
Br1—Mn1—N3—C10	22.5 (5)	C8—C9—C10—N3	−3.9 (12)
O1—Mn1—N4—C11	32.0 (5)	C15—N4—C11—C12	1.9 (11)
N3—Mn1—N4—C11	156.6 (8)	Mn1—N4—C11—C12	−176.9 (6)
N6—Mn1—N4—C11	−156.4 (5)	N4—C11—C12—C13	−3.0 (11)
N1—Mn1—N4—C11	117.6 (5)	C11—C12—C13—C14	1.4 (11)
Br1—Mn1—N4—C11	−63.8 (5)	C12—C13—C14—C15	1.3 (11)
O1—Mn1—N4—C15	−146.7 (5)	C11—N4—C15—N5	−179.1 (6)
N3—Mn1—N4—C15	−22.1 (12)	Mn1—N4—C15—N5	−0.5 (9)
N6—Mn1—N4—C15	24.9 (5)	C11—N4—C15—C14	0.9 (10)
N1—Mn1—N4—C15	−61.1 (5)	Mn1—N4—C15—C14	179.6 (5)
Br1—Mn1—N4—C15	117.5 (5)	C16—N5—C15—N4	−36.6 (11)
N4—Mn1—N6—C16	−33.8 (5)	C16—N5—C15—C14	143.3 (7)
N3—Mn1—N6—C16	135.8 (5)	C13—C14—C15—N4	−2.5 (10)
N1—Mn1—N6—C16	56.7 (5)	C13—C14—C15—N5	177.6 (6)
Br1—Mn1—N6—C16	−125.8 (5)	C20—N6—C16—N5	−175.2 (6)
N4—Mn1—N6—C20	158.8 (5)	Mn1—N6—C16—N5	17.7 (8)
N3—Mn1—N6—C20	−31.5 (5)	C20—N6—C16—C17	4.4 (10)
N1—Mn1—N6—C20	−110.7 (5)	Mn1—N6—C16—C17	−162.8 (5)
Br1—Mn1—N6—C20	66.8 (5)	C15—N5—C16—N6	26.8 (11)
C5—N1—C1—C2	−4.3 (10)	C15—N5—C16—C17	−152.8 (7)
Mn1—N1—C1—C2	142.2 (6)	N6—C16—C17—C18	−1.3 (10)
N1—C1—C2—C3	−3.1 (11)	N5—C16—C17—C18	178.3 (6)
C1—C2—C3—C4	7.3 (12)	C16—C17—C18—C19	−1.3 (10)
C2—C3—C4—C5	−4.4 (12)	C17—C18—C19—C20	0.8 (10)
C1—N1—C5—N2	−171.3 (6)	C16—N6—C20—C19	−5.0 (10)
Mn1—N1—C5—N2	44.1 (8)	Mn1—N6—C20—C19	163.2 (6)
C1—N1—C5—C4	7.5 (10)	C18—C19—C20—N6	2.5 (11)

### Hydrogen-bond geometry (Å, °)

**Table:**

*D*—H···*A*	*D*—H	H···*A*	*D*···*A*	*D*—H···*A*
O1—H1A···Br2^i^	0.84	2.50	3.304 (5)	160
O1—H1B···Br1^ii^	0.84	2.44	3.272 (5)	171
N2—H2N···Br2^iii^	0.92	2.62	3.472 (6)	154
N5—H5N···Br2^iv^	0.92	2.63	3.503 (6)	159

Symmetry codes: (i) *x*−1, *y*, *z*; (ii) −*x*, −*y*+1, −*z*+1; (iii) −*x*+1, −*y*+1, −*z*; (iv) *x*−1, *y*+1, *z*.
